# Expression and mechanistic roles of long non-coding RNAs in diabetic cataract: a systematic review and meta-analysis

**DOI:** 10.1186/s13062-026-00888-z

**Published:** 2026-08-12

**Authors:** Kai-Yang Chen, Hoi-Chun Chan, Chi-Ming Chan

**Affiliations:** 1 Department of General Medicine, Chang Gung Memorial Hospital (Linkou Branch), Taoyuan, Taiwan; 2 School of Pharmacy, China Medical University, Taichung, Taiwan; 3 Department of Ophthalmology, Cardinal Tien Hospital, New Taipei City, Taiwan; 4 School of Medicine, Fu Jen Catholic University, New Taipei City, Taiwan

**Keywords:** Long non-coding RNA, Diabetic cataract, Lens epithelial cells, MALAT1, GAS5, NEAT1, XIST, PVT1, RMRP, Epithelial-mesenchymal transition, Oxidative stress, M^6^A

## Abstract

**Introduction:**

Diabetic cataract (DC) is a lens-opacity complication of diabetes driven by hyperglycemia-related oxidative, apoptotic, metabolic, and epithelial–mesenchymal transition (EMT) pathways. This review evaluated the expression and mechanistic roles of long non-coding RNAs (lncRNAs) and lncRNA-related epitranscriptomic regulators in DC.

**Methods:**

PubMed, Embase, Cochrane Library, Scopus, Web of Science, and Google Scholar were searched to 1 June 2026 without language restriction. Ex vivo, in vitro, transcriptomic, epitranscriptomic, and mechanistic studies were included. Two reviewers screened records, extracted data, and assessed bias using the JBI checklist. Random-effects meta-analysis pooled Fisher-z-transformed correlations; bias and certainty were assessed using funnel plot, exploratory Egger’s test, and GRADE-adapted criteria.

**Results:**

Thirteen studies were included. Eight expression datasets showed a significant association between DC and lncRNA or lncRNA-linked epitranscriptomic dysregulation (pooled *r* = 0.445, 95% CI 0.338–0.541; *p* = 0.001), with low heterogeneity (I^2^ = 1.2%; Q = 7.088; df = 7; *p* = 0.42; τ^2^ = 0.00037; τ = 0.019; prediction interval 0.308–0.563). LINC01508, MAFA-AS1, MIAT, GAS5, XIST, MALAT1, KCNQ1OT1, and METTL16 were upregulated, whereas NEAT1 was downregulated. MALAT1, GAS5, XIST, PVT1, KCNQ1OT1, FOXD3–AS1, NEAT1, RMRP, METTL3, METTL16, FTO, METTL14, WTAP, ALKBH5, and YTHDF-family regulators converged on ceRNA signaling, apoptosis, oxidative stress, EMT, proliferation, mitochondrial dysfunction, ICAM-1 stabilization, and m^6^A-linked DKK1/Wnt/β-catenin regulation. Leave-one-out estimates ranged from *r* = 0.422 to 0.502; year-based meta-regression did not materially change the result.

**Conclusion:**

DC is associated with coordinated lncRNA and epitranscriptomic dysregulation across oxidative, apoptotic, EMT, mitochondrial, proliferative, and m^6^A-regulated pathways.

**Clinical trial number:**

Not applicable.

## Introduction

Diabetic cataract (DC) is an ocular complication of diabetes mellitus characterized by accelerated lens opacification under chronic hyperglycemic stress [1]. Cataract occurs earlier and more rapidly in diabetic people than in the general population, significantly contributing to the burden of visual disability around the world [2, 3]. Diabetes mellitus patients have three to five times higher cataract prevalence, with bilateral cataract being more frequent [4]. Additionally, cataracts develop at younger ages and progress more rapidly compared to non-diabetic individuals [5, 6]. DC has high psychosocial and economic costs, lowers the quality of life, and overburdens the healthcare system [7, 8]. With the increasing prevalence of diabetes, it is essential to understand the mechanisms of the onset and progression of DC.

DC has a complex, multifactorial pathophysiology that includes interconnected metabolic, oxidative, inflammatory, and apoptotic pathways [9]. The polyol pathway is stimulated by chronic hyperglycemia, leading to sorbitol accumulation and osmotic stress in lens epithelial cells [10]. Additionally, greater oxidative stress, caused by the production of reactive oxygen species, overwhelms the antioxidant defenses, leading to lipid peroxidation, protein glycation, and DNA damage [11, 12]. These metabolic imbalances stimulate the production of inflammatory cytokines and pro-apoptotic signaling cascades, leading to dysfunction of the lens epithelial cells and degeneration of the fibers [13]. Additionally, loss of lens transparency is caused by disruptions in calcium homeostasis, changes in cellular transport processes, and impaired autophagy [14]. These interconnected mechanisms are progressive, which makes DC a dynamic disease process that requires early intervention at the molecular level to prevent irreversible structural changes [15].

Existing management measures of DC include pharmacological and lifestyle interventions for glycemic control [16]. Proper metabolic control alone does not consistently stop the progression of cataracts [17]. Additionally, the use of topical agents and oral antioxidants has been investigated with considerable success [18]. Surgery has been the definitive therapy when there has been much opacification [19]. These interventions are usually undertaken once the lens damage is severe, when structural and functional degradation has already occurred [20]. The limited effective molecular-targeted preventive measures reflect an incomplete understanding of the early regulatory processes in DC pathogenesis [20]. Moreover, limited mechanistic understanding of the disease requires further studies of the molecular determinants of DC to discover new prevention and treatment options.

lncRNAs are a group of RNA transcripts longer than 200 nucleotides in size that have regulatory roles in a variety of biological processes, even though they are not protein-coding [21, 22]. LncRNAs regulate gene expression through different mechanisms, such as chromatin remodeling, transcriptional control, post-transcriptional processing, and epigenetic modification [23]. They act as molecular scaffolds, guides, decoys, and enhancers that regulate cell differentiation, proliferation, metabolic homeostasis, and apoptosis [24]. Additionally, they are associated with the pathogenesis of metabolic diseases such as diabetes mellitus and its complications [25]. Dysregulated lncRNA expression has been attributed to inflammation, fibrosis, and vascular dysfunction [26]. In ocular tissues, lncRNAs have been shown to regulate the behavior of lens epithelial cells, extracellular matrix remodeling, and oxidative-stress responses, making them potential components of the molecular landscape of DC [27]. Altered lncRNA expression is also reported in the lens anterior capsule in cataract associated with pathologic myopia [28], and NEAT1 dysregulation is implicated more broadly in type 2 diabetes mellitus and its complications [29].

Differentiated lncRNA expression patterns have been observed in diabetic lens tissues and cell models, with specific lncRNAs linked to oxidative stress responses, apoptosis, inflammation, and EMT [30–38]. Cataractogenic pathways can be modulated by certain lncRNAs, thereby affecting lens cell viability and transparency [39]. Heterogeneity in the reported effects and insufficient replication contrasts across independent cohorts require a structured synthesis of the role of lncRNAs in DC. This study critically evaluates the mechanistic and expression-based outcomes to reveal the functional relevance of lncRNAs and inform the development of new diagnostic and therapeutic procedures.

### Research purpose

The primary aim of this study is to evaluate the effects of lncRNAs in the development and progression of DC.

## Methodology

### Protocol and registration

This study was conducted adhering to the Preferred Reporting Items for Systematic Reviews and Meta-Analyses (PRISMA) statement [40]. The study protocol was prepared and registered with the International Prospective Register of Systematic Reviews (PROSPERO): CRD420261288418. The review followed the PRISMA 2020 main checklist, PRISMA abstract domains, and PRISMA flow-diagram framework to ensure transparent reporting of eligibility criteria, information sources, study selection, data extraction, synthesis methods, certainty assessment, and results.

### Search strategy for identifying studies

Two authors (K. Y. C., H. C. C.) developed the search strings using Medical Subject Headings (MeSH terms), Emtree terms, and keywords identified via PubMed and Google Search. Database search was conducted comprehensively across six primary sources: PubMed, Embase, Cochrane Library, Scopus, Web of Science Core Collection, and Google Scholar. ScienceDirect was not searched as a separate primary database. The search was run from each database’s inception to 1 June 2026. The search was then translated into the other databases. The comprehensive search strategies for all databases are reported in the supplemental materials. Google Scholar was searched using simplified keyword combinations, and results were screened by relevance. The first 200 results were reviewed, consistent with recommended methodological practice for systematic reviews. No language restrictions were applied, and non-English full texts were translated when necessary. Additionally, a manual search of other sources, including clinical trial registries such as ClinicalTrials.gov and the WHO International Clinical Trials Registry Platform, was conducted to identify ongoing or unpublished studies. The citations of the eligible studies were also searched to identify relevant articles to supplement the literature search results.

### Study selection

Two authors (K.Y.C, H.C.C) used Zotero (version 6.0.36) for study selection and screening. All retrieved records were imported, and the software algorithms automatically identified duplicates, which were then manually merged. Titles and abstracts were screened against eligibility criteria, and irrelevant studies were excluded. The remaining articles were then retrieved and evaluated for inclusion by accessing their full texts. Reasons for non-inclusion at the full-text level were recorded. They conducted the screening independently and, in cases of disagreement, resolved them through discussion. Unresolved disagreement was adjudicated by a third reviewer (C. M. C).

### Eligibility criteria

Articles were included in the present study when they met the following eligibility criteria according to the PICOS criteria [41].

### Inclusion criteria

Population included human diabetic cataract tissues, anterior lens capsule samples, diabetic cataract-derived lens epithelial cells, and human lens epithelial cell lines exposed to high glucose. The intervention or exposure of interest included dysregulated lncRNA expression, lncRNA knockdown or overexpression, lncRNA-miRNA-mRNA axis manipulation, or upstream epitranscriptomic regulators involving lncRNA pathways. Eligible comparators included non-diabetic cataract tissue, age-related cataract tissue, normal lens epithelial tissue, normal-glucose-treated lens epithelial cells, and matched negative-control transfection groups. Outcomes were predefined according to primary and secondary categories. Primary outcomes included lncRNA expression difference, apoptosis, oxidative stress, EMT, and proliferation/viability. Secondary outcomes included migration, invasion, pathway activation, miRNA/mRNA target validation, rescue-experiment evidence, human-tissue validation, and m^6^A-related regulatory findings. Eligible study designs included peer-reviewed original ex vivo, in vitro, transcriptomic, epitranscriptomic, and mechanistic experimental studies. There was no language restriction.

### Exclusion criteria

The study excluded review articles, conference abstracts, meeting abstracts, posters, proceedings-only records, preprints without peer-reviewed publication, systematic reviews, meta-analyses, editorials, commentaries, letters, case reports, study protocols, and non-peer-reviewed publications. Animal studies and studies not involving diabetic cataract were excluded. Studies were excluded when they did not investigate diabetic cataract tissue, diabetic cataract-derived cells, or a high-glucose lens epithelial cell model, or when diabetic cataract-specific data could not be separated. Additionally, studies with insufficient data for extraction, duplicate publications, and studies evaluating ocular diseases unrelated to DC without separate DC-specific analyses were excluded from the review.

### Risk of bias assessment

Two reviewers (K. Y. C., H. C. C) conducted quality appraisal independently using the JBI Critical Appraisal Checklist for Quasi-Experimental Studies [42]. Disagreements were resolved by discussion between the two reviewers or with a third reviewer (C. M. C.) when necessary.

Risk of bias was assessed using the JBI Critical Appraisal Checklist for Quasi-Experimental Studies. Each study was evaluated across nine methodological domains and rated as “Yes,” “No,” “Unclear,” or “NA” for each criterion. Overall study quality was appraised as “Include,” “Exclude,” or “Seek Further Information” in accordance with JBI recommendations. The item-level judgment criteria used to interpret the JBI Critical Appraisal Checklist are summarized in Table 1, and the study-level risk-of-bias assessment results are presented in Table 2.

**Table 1:** JBI item level judgement

JBI Appraisal Item	Criteria Met (Yes)	Unclear	Criteria Not Met (No)
Q1: Cause-and-effect relationship	The intervention/exposure clearly precedes the outcome and the relationship is well described.	Temporal sequence is incompletely described.	Cause and effect are unclear or cannot be established.
Q2: Similarity of participants	Comparison groups are similar and baseline characteristics are adequately reported.	Baseline characteristics incompletely reported.	Important baseline differences likely influence outcomes.
Q3: Similarity of treatment/care	Groups received similar care apart from the intervention/exposure of interest.	Minor differences in care or experimental conditions.	Major differences in treatment or care likely affect outcomes.
Q4: Control group	Appropriate control/comparator group included.	Control group inadequately described.	No control group or inappropriate comparator.
Q5: Multiple outcome measurements	Outcomes assessed at multiple relevant time points when appropriate.	Timing of measurements incompletely reported.	Outcome assessment insufficient to establish intervention effects.
Q6: Completeness of follow-up	Follow-up complete or missing data adequately addressed.	Missing data/follow-up inadequately reported.	Substantial missing data likely to introduce bias.
Q7: Consistency of outcome measurement	Outcomes measured identically across comparison groups.	Minor uncertainty regarding measurement consistency.	Different outcome assessment methods used across groups.
Q8: Reliability of outcome measurement	Validated and reliable measurement methods used.	Reliability incompletely reported.	Outcome measurements poorly described or unreliable.
Q9: Statistical analysis	Appropriate statistical analyses used and adequately reported.	Statistical methods incompletely described.	Inappropriate statistical analyses or major reporting deficiencies.
Overall Appraisal	Most criteria fulfilled; study suitable for inclusion (Include).	Additional information required or one or more important criteria unclear (Seek Further Information).	Several criteria not fulfilled and validity substantially compromised (Exclude).

**Table 2:** JBI assessment risk of bias assessment results

Study ID	Q1	Q2	Q3	Q4	Q5	Q6	Q7	Q8	Q9	Overall
[43]	Yes	Yes	Yes	Yes	No	NA	Yes	Unclear	No	Include
[30]	Yes	Yes	Yes	Yes	No	NA	Yes	Yes	Yes	Include
[31]	Yes	Unclear	Yes	Yes	No	NA	Yes	Yes	Unclear	Include
[32]	Yes	Yes	Yes	Yes	No	NA	Yes	Yes	Yes	Include
[33]	Yes	Yes	Yes	Yes	No	NA	Yes	Yes	Yes	Include
[34]	Yes	Yes	Yes	Yes	No	NA	Yes	Unclear	No	Include
[35]	Yes	Yes	Yes	Yes	No	NA	Yes	Yes	Yes	Include
[36]	Yes	Unclear	Unclear	Yes	No	NA	Yes	Yes	Yes	Include
[44]	Yes	Yes	Yes	Yes	No	NA	Yes	Yes	Yes	Include
[37]	Yes	Yes	Yes	Yes	No	NA	Yes	Unclear	Yes	Include
[38]	Yes	Yes	Yes	Yes	No	NA	Yes	Yes	Yes	Include
[45]	Yes	Yes	Yes	Yes	No	NA	Yes	Unclear	No	Include
[46]	Yes	Yes	Yes	Yes	No	NA	Yes	Unclear	No	Include

### Outcome and effect measures

Outcomes were prespecified and categorized as primary and secondary outcomes to ensure methodological consistency.

### Primary outcomes

Primary outcomes included lncRNA expression difference, apoptosis, oxidative stress, EMT, and proliferation/viability. lncRNA expression was synthesized as pooled correlation when the original expression statistics were compatible with correlation-based modeling. Continuous experimental outcomes were planned for synthesis using standardized mean differences when sufficient mean, SD, and sample-size data were available.

### Secondary outcomes

Secondary outcomes included migration, invasion, pathway activation, miRNA/mRNA target validation, rescue-experiment evidence, human-tissue validation, and m^6^A-related regulatory findings. Pathway activation and target validation were summarized qualitatively when assay platforms, reporting formats, or molecular targets differed across studies.

### Data selection and extraction

A data extraction template was developed using Microsoft Excel 2021. Two reviewers (K. Y. C., H. C. C) independently extracted the study characteristics, including study ID, study design, country, sample type, age (years), sample size, patient characteristics, cell line, comparator type, glucose concentration, lncRNA studied, mechanism / regulatory axis, method of lncRNA analysis, outcomes available for meta-analysis, and main findings. Extracted data were cross-checked, and disagreements were resolved by discussion and adjudication by a third reviewer (C. M. C.) when necessary.

### Handling missing data and data transformation

Missing data were sought from supplementary materials and related publications when available. When outcome data, sample characteristics, or methodological details could not be obtained, the available information was extracted and reported qualitatively. No imputation of missing outcome data was performed. Studies with insufficient data for quantitative synthesis were retained in the qualitative review where relevant. The potential impact of missing information on study interpretation was considered during risk-of-bias assessment and evidence certainty evaluation.

Data were extracted in their original reported format. Means and SDs were preferentially extracted directly; SEs, SEMs, CIs, interquartile ranges, ranges, fold-change statistics, and graphically reported values were converted only when the required transformation was mathematically defined and the direction of effect was unambiguous. Probability-only statements such as *p* < 0.05 or *p* < 0.01 were not used to reconstruct expression magnitude. Studies with insufficient quantitative data were included in the qualitative synthesis only. Outcomes were grouped into predefined thematic categories and synthesized narratively. No imputation of missing data was performed.

### Data analysis

The qualitative data extracted were synthesized using a structured thematic analysis. It was done through a step-by-step process, and it included familiarizing with the data, creating codes, sorting into possible themes, polishing and validating themes, defining and naming each theme, and writing a narrative synthesis [47].

### Meta-analysis

A research author (C. M. C.) used CMA software to conduct meta-analyses [48]. Quantitative synthesis was performed using the available expression statistics reported by the included studies. For the expression meta-analysis, Fisher-z-transformed correlations were pooled under a random-effects model and then back-transformed to correlations for reporting. Non-lncRNA m^6^A-regulator studies were interpreted as epitranscriptomic modules rather than direct lncRNA expression studies unless the extracted effect explicitly represented an lncRNA target.

Where quantitative pooling was feasible, pooled correlations and corresponding 95% CIs were calculated using a random-effects model, reflecting methodological and biological heterogeneity across studies. Qualitative synthesis was undertaken for mechanistic outcomes, including oxidative stress, apoptosis, epithelial-mesenchymal transition (EMT), proliferation, migration, mitochondrial dysfunction, and m^6^A-related epitranscriptomic regulation.

Statistical heterogeneity was assessed using Cochran’s Q test and quantified using the I^2^ statistic, τ^2^, τ, and 95% prediction intervals. Heterogeneity was interpreted according to Cochrane recommendations, with I^2^ values of 0–40% considered low, 30–60% moderate, 50–90% substantial, and 75–100% considerable heterogeneity. Statistical significance was defined as a two-sided *p*-value < 0.05.

Forest plots were generated to visualize pooled effect estimates and study-specific results. Sensitivity analyses included leave-one-out analysis, fixed-effect versus random-effects comparison, and exclusion-based checks for studies without direct lncRNA expression targets. Reporting bias was assessed using funnel-plot inspection and exploratory Egger’s test when the quantitative synthesis contained a sufficient number of datasets for descriptive interpretation. Exploratory univariable meta-regression was prespecified for publication year, human-tissue validation, cell line, comparator type, glucose concentration, assay platform, and risk-of-bias score when the number of independent effect estimates supported stable modeling.

## Results

### Study selection and screening

**Fig. 1:**
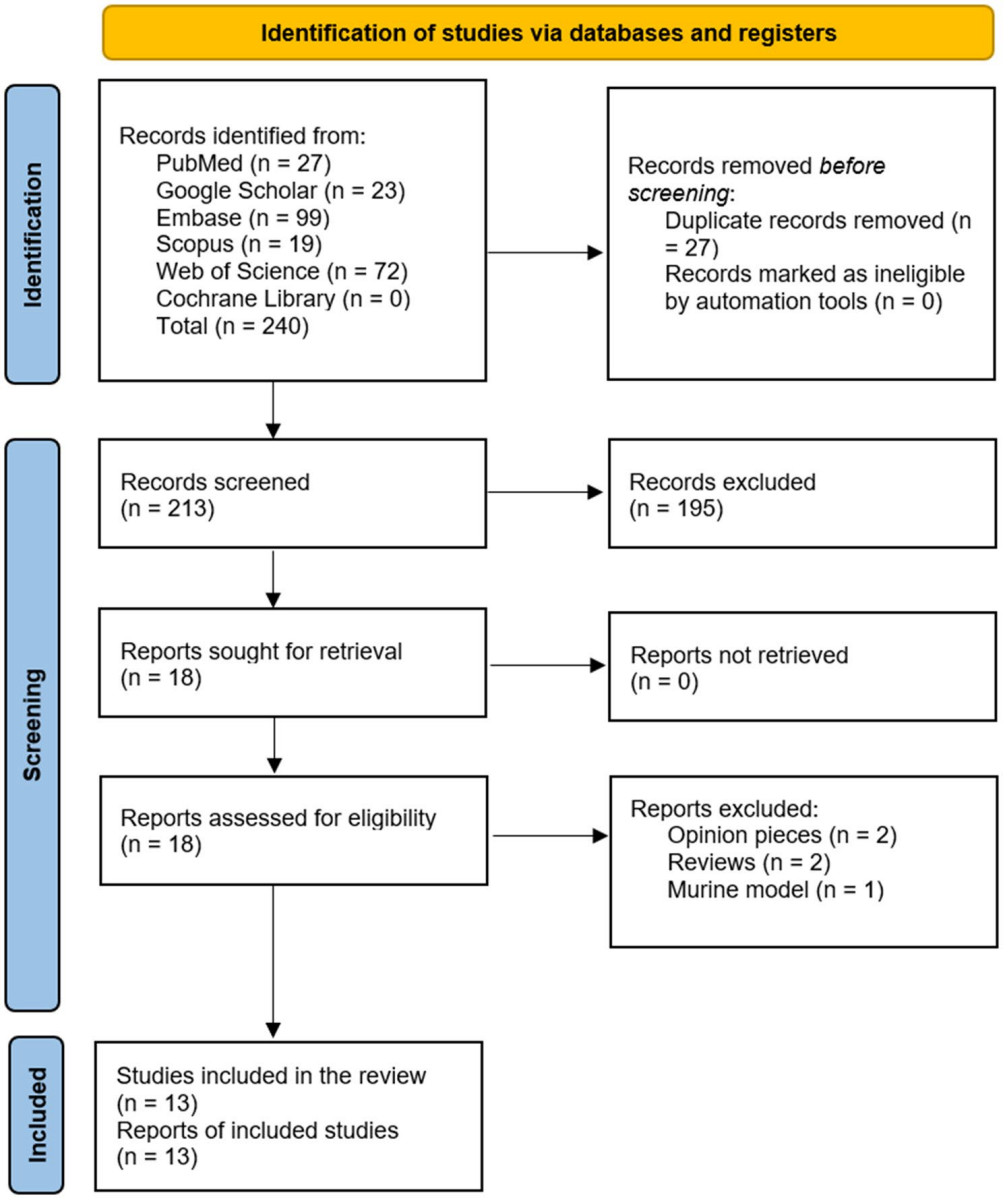
PRISMA flow diagram [40]

The electronic database search yielded 240 records, of which 27 were duplicates. Furthermore, 195 records were removed based on titles and abstracts. The remaining 18 were then retrieved for full-text assessment; after which, five were excluded, leaving 13 articles included in this study (Fig. 1) [30–38, 43–46].

### Risk of bias assessment

The risk of bias assessment evaluated 13 experimental studies across nine methodological criteria, including causality clarity, participant comparability, similarity of treatment or care, control groups, multiple outcome measurements, completeness of follow-up, outcome measurement consistency, measurement reliability, and statistical analysis appropriateness. Most studies fulfilled the majority of JBI criteria and were considered suitable for inclusion, with several studies demonstrating unclear reporting or specific methodological domains requiring careful interpretation [30–38, 43–46]. This assessment demonstrates that the included studies used clear experimental designs, appropriate controls, consistent outcome measurements, and interpretable statistical approaches (Table 2).

### Overview of study characteristics

This analysis included 13 studies investigating lncRNAs and lncRNA-related epitranscriptomic regulation in DC, conducted in China and published between 2018 and 2026. Studies employed ex vivo human lens epithelial tissue from patients with T2DM and in vitro cell culture models under high-glucose conditions. Studies examined various lncRNAs, including MALAT1, NEAT1, PVT1, XIST, MIAT, KCNQ1OT1, FOXD3–AS1, RMRP, and lncRNA-linked m^6^A regulators, including METTL3, METTL16, METTL14, WTAP, FTO, ALKBH5, and YTHDF family members [30–38, 43–46]. The studies investigated cell apoptosis, proliferation, oxidative stress markers, EMT, migration, mitochondrial dysfunction, and epitranscriptomic regulation (Table 3).

**Table 3:** Study characteristics

Study ID	Study Design	Country	Sample Type	Age (years)	Sample Size	Patient Characteristics	Cell Line	Comparator Type	Glucose Concentration	lncRNA Studied	Mechanism / Regulatory Axis	Method of lncRNA Analysis	Outcomes Available for Meta-analysis	Main Findings
[43]	In vitro experimental	China	HLEB3 cells	NR	n = 3	NR	HLEB3	NG-treated cells	40 mM	FOXD3–AS1	FOXD3–AS1/miR-338-3p	RT-qPCR, luciferase assay	Expression, apoptosis, oxidative stress	FOXD3–AS1 knockdown alleviated HG-induced oxidative stress and apoptosis.
[30]	Ex vivo + in vitro experimental	China	Human lens tissues and SRA01/04 cells	62–69	NR	T2DM; FBG 7.99 ± 0.53 mmol/L	SRA01/04	Normal lenses; NG-treated cells	25 mM	MALAT1	SP1/MALAT1/p38MAPK	qRT-PCR	Expression, apoptosis, oxidative stress, viability	MALAT1 promoted oxidative stress and apoptosis through p38MAPK activation.
[31]	Ex vivo + in vitro observational experimental	China	Lens epithelium and SRA01/04 cells	62–68	Tissue *n* = 3	T2DM; HbA1c 7.5–7.9%	SRA01/04	Normal eyes; ARC	HG (NR)	LINC01508, MAFA-AS1, MIAT, IGFBP7–AS1, AHCTF1P1, DARS-AS1, LOC103171574, SP2–AS1	Transcriptomic dysregulation pathways	Microarray, qRT-PCR, GO/KEGG	Expression	Differentially expressed lncRNAs associated with glucose metabolism and oxidative stress.
[32]	Ex vivo + in vitro experimental	China	Human lens tissues and HLE B-3 cells	NR	NR	T2DM	HLE B-3	Normal eyes; NG-treated cells	HG (NR)	NEAT1	YY1/NEAT1/miR-205-3p/MMP16	qRT-PCR, ChIP, luciferase assay	Expression, proliferation, apoptosis	Reduced NEAT1 promoted apoptosis and inhibited proliferation.
[33]	Ex vivo + in vitro observational experimental	China	Human lens epithelial cells	NR	n = 3 replicates	Grade III cortical cataract; T2DM	LECs, 293T	ARC; NG-treated cells	HG (NR)	GAS5	GAS5/miR-204-3p/TGFBR1/Smad2	qRT-PCR	Expression, EMT, migration	GAS5 promoted EMT through TGFBR1 activation.
[34]	Ex vivo + In vitro experimental	China	Human lens epithelial tissues and cultured cells	NR	NR	Diabetic cataract	Human lens epithelial cells	Normal lens tissues; NG-treated cells	HG (NR)	KCNQ1OT1	KCNQ1OT1/miR-26a-5p/ITGAV/TGF-β/Smad3	RT-qPCR, luciferase assay, functional assays	Expression, proliferation, migration, EMT	KCNQ1OT1 knockdown inhibited viability, migration, and EMT in diabetic cataract lens epithelial cells.
[36]	Ex vivo + in vitro observational experimental	China	Human lens tissues and SRA01/04 cells	52–60	n = 3 replicates	DC patients	SRA01/04	Normal eyes	HG (NR)	XIST	XIST/miR-34a/SMAD2	qRT-PCR, RIP, luciferase assay	Expression, proliferation, migration, EMT	XIST promoted proliferation, migration, and EMT.
[44]	Ex vivo + in vitro observational experimental	China	Human lens tissues and HLE B-3 cells	NR	NR	DC patients	HLE B-3	Normal eyes; NG-treated cells	HG (NR)	m^6^A-related regulators	METTL3/ICAM-1	m^6^A profiling, expression analysis	Expression, apoptosis, proliferation	METTL3-mediated m^6^A modification promoted lens cell damage.
[37]	Ex vivo + in vitro observational experimental	China	Human lens tissues and HLE B-3 cells	NR	NR	DC patients	HLE B-3	Normal eyes; NG-treated cells	HG (NR)	PVT1	SP1/PVT1/miR-214-3p/MMP2	qRT-PCR, FISH, ChIP	Expression, apoptosis, proliferation	PVT1 promoted apoptosis and reduced proliferation.
[38]	Ex vivo + in vitro observational experimental	China	Human lens tissues and HLE B-3 cells	64.94 ± 9.72	NR	DC patients	HLE B-3	ARC; NG-treated cells	HG (NR)	MALAT1	MALAT1/miR-144-3p/NRF2/Notch1/Snail	qRT-PCR, luciferase assay	Expression, oxidative stress, EMT, migration	MALAT1 promoted EMT, migration, and oxidative stress.
[45]	Ex vivo observational	China	Lens epithelial tissues	NR	NR	T2DM > 8 years	None	Normal eyes	NA	m^6^A regulators	METTL3/METTL14/WTAP/FTO axis	MeRIP-seq, scRNA-seq	Expression	Increased m^6^A modification and upregulation of core m^6^A regulators in DC.
[46]	Ex vivo + In vitro experimental	China	Human lens epithelial tissues and cultured cells	NR	NR	Diabetic cataract	Human lens epithelial cells	Normal controls; NG-treated cells	HG (NR)	NR (m^6^A-associated targets)	METTL16/DKK1/Wnt/β-catenin	RT-qPCR, m^6^A analysis, functional assays	Proliferation, apoptosis, expression	METTL16 regulated lens epithelial cell dysfunction through m^6^A-modified DKK1 signaling.
[35]	Ex vivo + in vitro	China	Human lens tissue; HLECs	NR	NR	DC patients	HLECs	Normal controls; NG-treated cells	HG (NR)	RMRP / FTO m^6^A axis	FTO/RMRP mitochondrial axis	RT-qPCR; m^6^A; mitochondrial assays	Expression; mitochondrial function	FTO/RMRP regulated mitochondrial dysfunction in DC.

To move beyond a simple list of dysregulated lncRNAs, the evidence was organized into pathway-level modules. Included studies clustered around oxidative stress and apoptosis, EMT/fibrotic remodeling, proliferation and survival, inflammatory adhesion, transcriptomic dysregulation, and m^6^A-related epitranscriptomic regulation. This framework shows that lncRNAs in diabetic cataract do not act as isolated markers, but as components of interconnected regulatory networks affecting lens epithelial cell injury under hyperglycemic stress. To move beyond a simple list of dysregulated lncRNAs, the evidence was organized into pathway-level modules. The pathway-level evidence map of lncRNAs and lncRNA-linked epitranscriptomic regulators in diabetic cataract is summarized in Table 4.

**Table 4:** Pathway-level evidence map of long non-coding RNAs in DC

Mechanistic module	Included lncRNAs/regulators	Main regulatory axis	Functional implication
Oxidative-stress/apoptosis	MALAT1, FOXD3–AS1, PVT1, NEAT1, METTL3, RMRP/FTO	MALAT1/p38MAPK; FOXD3–AS1/miR-338-3p; PVT1/miR-214-3p/MMP2; NEAT1/miR-205-3p/MMP16; METTL3/ICAM-1; FTO/RMRP	Regulates ROS accumulation, MDA, SOD/CAT/GSH-Px activity, caspase activation, Bax/Bcl-2 balance, mitochondrial dysfunction, and HG-induced lens epithelial cell apoptosis.
EMT/fibrosis and migration	GAS5, MALAT1, XIST, NEAT1, PVT1	GAS5/miR-204-3p/TGFBR1/Smad2; MALAT1/miR-144-3p/NRF2/Notch1/Snail; XIST/miR-34a/SMAD2; NEAT1/MMP16; PVT1/MMP2	Promotes loss of epithelial phenotype, increased α-SMA, vimentin, N-cadherin, migration, invasion, and lens epithelial remodeling.
Proliferation/survival	NEAT1, XIST, PVT1, METTL3, MALAT1	YY1/NEAT1; XIST/miR-34a/SMAD2; SP1/PVT1; METTL3/ICAM-1; MALAT1-related cell-cycle regulation	Alters viability, proliferation, EdU/CCK-8/MTT outcomes, and survival of lens epithelial cells under high-glucose stress.
Inflammatory adhesion	METTL3-related m^6^A pathway	METTL3-mediated m^6^A modification of ICAM-1 mRNA	Stabilizes ICAM-1 expression and links diabetic cataract to inflammatory adhesion and cellular injury.
m^6^A-epitranscriptomic module	METTL3, METTL14, WTAP, FTO, METTL16, RMRP	m^6^A methylation landscape, METTL3/ICAM-1, METTL16/DKK1/Wnt/β-catenin, and FTO/RMRP signaling in diabetic cataract lens epithelial cells	Indicates higher-order post-transcriptional regulation beyond individual ceRNA axes, affecting pathogenic mRNA stability, mitochondrial function, Wnt signaling, and cataract severity.

### Integrated lncRNA–miRNA–mRNA regulatory network in diabetic cataract

**Fig. 2:**
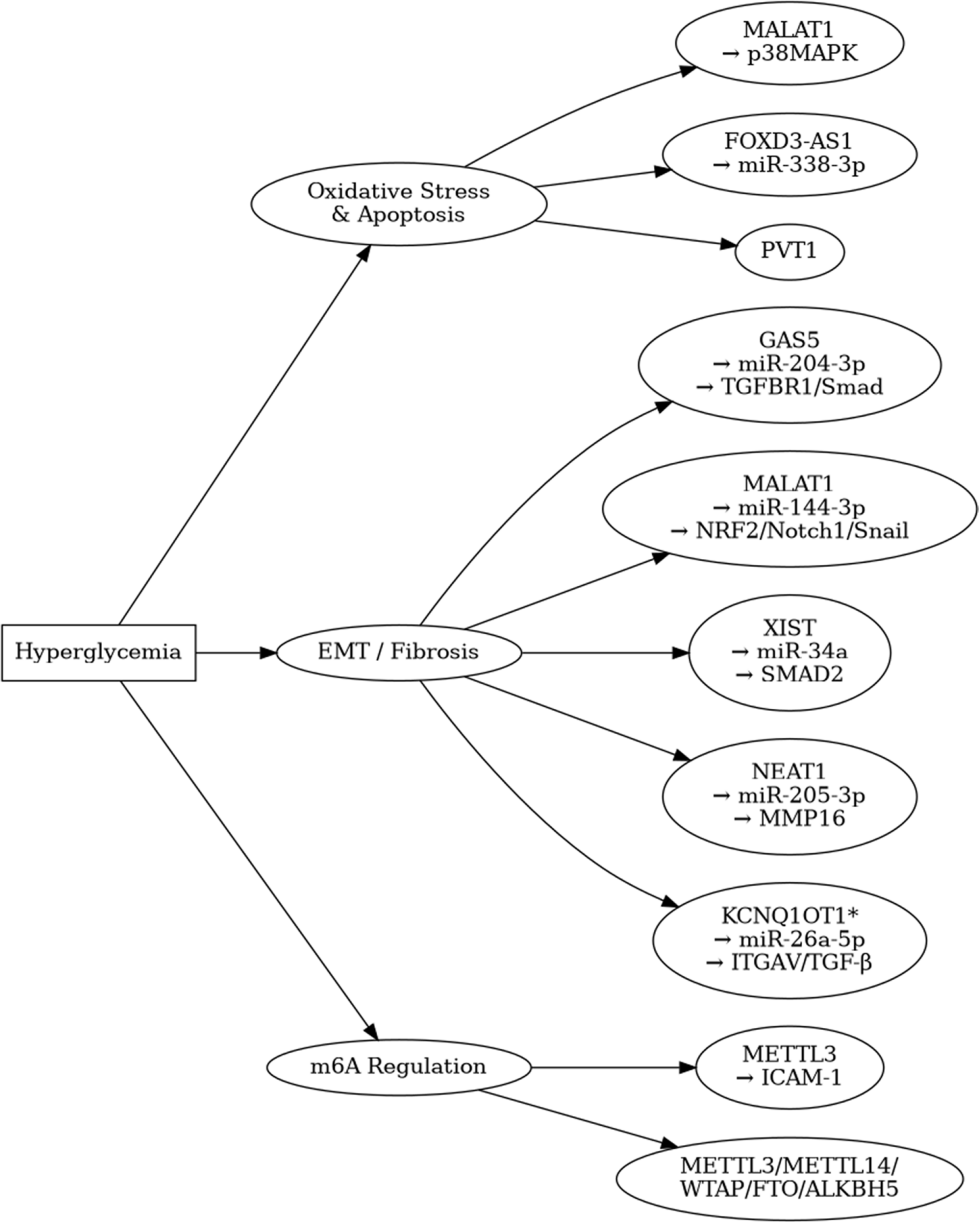
Integrated lncRNA–miRNA–mRNA regulatory network in DC

Hyperglycemia induces dysregulation of multiple lncRNAs that converge on five major pathogenic modules: oxidative stress and apoptosis involving MALAT1, FOXD3–AS1, PVT1, NEAT1, and METTL3; EMT and fibrotic remodeling involving GAS5, MALAT1, XIST, PVT1, NEAT1, and KCNQ1OT1; proliferation and survival involving XIST, NEAT1, PVT1, and MALAT1; inflammatory adhesion involving the METTL3-ICAM-1 pathway; and m^6^A epitranscriptomic regulation involving METTL3, METTL14, WTAP, FTO, METTL16, RMRP, ALKBH5, and YTHDF family members. Together, these pathways contribute to lens epithelial cell injury and diabetic cataract progression (Fig. 2).

### Translational-readiness table

The translational readiness of candidate lncRNAs and lncRNA-linked epitranscriptomic regulators, including human-tissue validation, functional rescue evidence, pathway validation, replication status, biomarker potential, and therapeutic potential, is summarized in Table 5.

**Table 5:** Translational-readiness table

lncRNA / Regulator	Direction of Change	Sample Type	Human Tissue Validation	Functional Rescue Experiment	Pathway Validation	Replication Status	Biomarker Potential	Therapeutic Potential
MALAT1	Upregulated	Human lens tissue + HLECs	Yes	Yes	Yes; p38MAPK and miR-144-3p/NRF2/Notch1/Snail	Replicated in ≥ 2 studies	Moderate	Moderate
GAS5	Upregulated	Human LECs + cell model	Yes	Yes	Yes; miR-204-3p/TGFBR1/Smad	Single study	Low–moderate	Moderate
XIST	Upregulated	Human lens tissue + SRA01/04	Yes	Yes	Yes; miR-34a/SMAD2	Single study	Low–moderate	Moderate
PVT1	Upregulated	Human lens tissue + HLE B-3	Yes	Yes	Yes; miR-214-3p/MMP2	Single study	Low–moderate	Moderate
NEAT1	Downregulated	Human lens tissue + HLE B-3	Yes	Yes	Yes; miR-205-3p/MMP16	Single study	Low	Moderate
FOXD3–AS1	Upregulated under high-glucose conditions; knockdown was protective in one in vitro study	HLEB3 cells	No/unclear	Yes	Yes; miR-338-3p	Single in vitro study	Low	Low–moderate
LINC01508	Upregulated	Human lens tissue + SRA01/04	Yes	No	Co-expression/pathway prediction	Single transcriptomic study	Low	Low
MAFA-AS1	Upregulated	Human lens tissue + SRA01/04	Yes	No	Co-expression/pathway prediction	Single transcriptomic study	Low	Low
MIAT	Upregulated	Human lens tissue + SRA01/04	Yes	No	Co-expression/pathway prediction	Single transcriptomic study	Low	Low
KCNQ1OT1	Upregulated	Human lens tissue + HLECs	Yes	Yes	Yes; miR-26a-5p/ITGAV/TGF-β/Smad	Single mechanistic study	Potentially moderate	Potentially moderate
METTL3 / m^6^A axis	Upregulated	Human lens tissue + HLE B-3	Yes	Yes	Yes; m^6^A/ICAM-1	≥2 m^6^A studies	Low–moderate	Moderate
METTL14 / WTAP / FTO / ALKBH5 / YTHDF	Dysregulated	Human lens tissue	Yes	No/unclear	m^6^A landscape validation	Single study	Low	Low
RMRP / FTO m^6^A axis	Dysregulated	Human lens tissue + HLECs	Yes	Yes	Yes; FTO-mediated m^6^A demethylation and mitochondrial dysfunction	Single mechanistic study	Low-moderate	Moderate
METTL16 / DKK1 axis	Upregulated	Human lens tissue + HLECs	Yes	Yes	Yes; DKK1/Wnt/β-catenin signaling	Single mechanistic study	Low-moderate	Moderate

### Certainty of evidence

A structured GRADE-adapted certainty-of-evidence assessment was performed for all prespecified primary and secondary outcomes. The assessment considered risk of bias, inconsistency, indirectness, imprecision, and reporting bias in the context of experimental diabetic cataract evidence. Certainty judgments are summarized in Table 6 and align the quantitative expression result with the mechanistic evidence across apoptosis, oxidative stress, EMT, proliferation/viability, migration, target validation, mitochondrial dysfunction, and m^6^A-related regulation.

**Table 6:** Structured certainty-of-evidence assessment for primary and secondary outcomes

Outcome domain	Evidence base	No. of studies/datasets	Direction and synthesis	Certainty	Rationale
lncRNA and lncRNA-linked epitranscriptomic target expression	Human tissue and high-glucose HLEC expression datasets	8 datasets	Pooled correlation *r* = 0.445 (95% CI: 0.338 to 0.541), *p* = 0.001; direction-specific interpretation for upregulated lncRNAs and downregulated NEAT1	Moderate	Consistent quantitative direction across most datasets with low statistical heterogeneity and direct DC-related sample sources.
Apoptosis	Functional experiments assessing apoptotic rate, TUNEL, caspase, Bax/Bcl-2, or related markers	Multiple mechanistic studies	MALAT1, PVT1, METTL3, XIST, NEAT1, and FOXD3–AS1 axes converged on apoptosis regulation under HG exposure	Low-moderate	Mechanistic convergence across independent assays and lncRNA-specific pathways supports biological coherence.
Oxidative stress	ROS, MDA, SOD, CAT, GSH-Px, and redox pathway assays	Several mechanistic studies	MALAT1 and FOXD3–AS1 showed reproducible links with oxidative-stress regulation in HG-treated lens epithelial cells	Low-moderate	Consistent redox-marker direction within experimental systems and direct alignment with DC pathophysiology.
EMT and migration	EMT marker, wound-healing, Transwell, TGF-β/Smad, Notch/Snail, and MMP-related assays	Multiple mechanistic studies	GAS5, MALAT1, XIST, PVT1, NEAT1, and KCNQ1OT1 converged on EMT, migration, invasion, and matrix-remodeling pathways	Low-moderate	Pathway validation by luciferase, ChIP, RIP, knockdown, and rescue approaches strengthened mechanistic interpretation.
Proliferation/viability	CCK-8, MTT, EdU, cell-cycle, and viability assays	Multiple mechanistic studies	XIST, PVT1, NEAT1, METTL3, KCNQ1OT1, MALAT1, and METTL16 affected survival and proliferative activity under HG conditions	Low-moderate	Multiple functional readouts supported lens epithelial cell phenotype regulation.
m^6^A-related regulation and mitochondrial dysfunction	m^6^A profiling, MeRIP-seq, regulator expression, target validation, and mitochondrial assays	Several epitranscriptomic studies	METTL3/ICAM-1, METTL16/DKK1/Wnt/β-catenin, FTO/RMRP, and METTL14/WTAP/FTO modules supported epitranscriptomic control in DC	Low-moderate	Epitranscriptomic modules expanded the review beyond ceRNA axes and connected lncRNA regulation with RNA methylation and mitochondrial function.

Certainty labels in this table represent an author-defined descriptive framework adapted from GRADE principles and do not represent formal GRADE certainty ratings, since “Low-moderate” is not one of the four defined GRADE categories (High, Moderate, Low, Very low)

## Systematic review results

### Specific LncRNAs and their regulatory axes in DC pathogenesis

The lens epithelial abnormalities in DC are frequently initiated by abnormal levels of long non-coding RNAs, resulting from high blood sugar-induced changes in the activity of certain transcription factors [30, 32, 37]. The transcription factor SP1 is upregulated under high-glucose conditions and binds the promoter of the lncRNA MALAT1, thereby increasing its synthesis [30]. Similarly, SP1 activates transcription of the lncRNA PVT1 by binding to its promoter [37]. In contrast, transcription factor YY1 was decreased in DC, and as a result, the activation of the target lncRNA NEAT1 decreases [32].

Dysregulated lncRNAs contribute to pathogenic lens epithelial cell phenotypes and enhance oxidative stress, significantly contributing to cataract formation [30, 36, 43]. A higher concentration of MALAT1 via the SP1–MALAT1 pathway activates p38 MAPK signaling, inducing cell death and increasing oxidative stress in lens epithelial cells [30]. Similarly, XIST increases cell damage, activates its target SMAD2, induces cell proliferation and migration, and decreases apoptosis in lens epithelial cells [36]. In another study, PVT1 is upregulated in the lens epithelial cell and contributes to cell death under diabetic stress through its isolated pathway, affecting downstream targets [37]. Knockdown of lncRNA FOXD3–AS1 preserves epithelial cells by up-regulating miR-338-3p, which reduces high-glucose-associated oxidative stress and cell death [43]. Additionally, the downregulation of lncRNA NEAT1 under high-glucose conditions suppresses LEC proliferation and induces apoptosis [32].

### EMT

EMT is regulated through unique lncRNA competing RNA pathways. Elevated lncRNA GAS5 traps miR-204-3p and elevates the levels of the TGFBR1 and Smad2 pathway [33]. Consequently, this process increases EMT and cell movement [33], and MALAT1 also promotes EMT and migration by binding to miR-144-3p, thereby lifting inhibition of NRF2 and activating the Notch1-Snail pathway [38]. XIST remodels tissues by disrupting normal tissue structure, facilitating the migration and invasion of lens epithelial cells [36]. Matrix metalloproteinase-related axes alter lens epithelial remodeling and augment cell invasion [32, 37]. The inactivation of the NEAT1 miR-205-3p pathway results in decreased MMP16 levels, which is involved in cell dysfunction [32]. In addition, the SP1–PVT1 pathway amplifies the effect of MMP2 through miR-214-3p and affects lens epithelial cell growth and survival under diabetic conditions [37]. Moreover, the lncRNA MALAT1 can enhance cell invasion by regulating cell movement through its modulation of the NRF2, Notch1, and Snail pathways. Additionally, it increases the invasiveness of lens epithelial cells [38].

### Functional roles and mechanisms of LncRNAs in lens epithelial cell dysfunction

Abnormal lncRNA expression in DC disrupts cellular homeostasis, leading to programmed cell death and oxidative injury [30, 43]. MALAT1 overexpression under HG conditions promoted pro-apoptotic indices (Bax, Caspase-3), as well as indices of oxidative stress (Malondialdehyde, MDA), but suppressed anti-apoptotic (Bcl-2) and antioxidant enzymes (Superoxide Dismutase, SOD; Glutathione Peroxidase, GSH-Px), mediated by the activation of the p38MAPK signaling pathway [30]. Moreover, MALAT1 also modulates ROS, as its downregulation suppressed HG-mediated ROS accumulation in HLE-B3 cells [38]. In contrast, NEAT1 functions as a protective mediator, with its downregulation under HG conditions increasing apoptosis, reducing proliferation, and injuring lens epithelial cells [32]. Similarly, FOXD3-AS1 knockdown also reduced apoptosis, cleaved caspase-3/9, as well as HG-mediated oxidative injury by increasing the activity of antioxidant enzymes (SOD, CAT), while reducing lipid peroxides (MDA) [43]. Moreover, PVT1 knockdown reduced the HG-mediated increase in apoptotic index assessed by flow cytometry [37], while XIST gene depletion also induced apoptosis in stimulated HG cells, as assessed by TUNEL assay [36].

DC is a process of pathological tissue remodeling, with lncRNAs playing a critical role by reducing epithelial marker expression and promoting cellular motility [32, 38]. The knockdown of GAS5 reduced HG-induced EMT, with a restoration of E-cadherin, a reduction in mesenchymal proteins (N-cadherin, Vimentin, and α-SMA), and a decrease in cell migration in scratch assays, as well as decreased Smad2 phosphorylation, a central mediator of the TGF-β signaling pathway that induces EMT [33]. MALAT1 knockdown blocks HG-induced expression of mesenchymal proteins (Fibronectin and α-SMA), thereby restoring E-cadherin and ZO-1. MALAT1 knockdown also decreased HG-induced cellular migration in Transwell and wound-healing assays [38]. The knockdown of XIST also blocked HG-induced cellular migration (wound-healing test) and invasion (Transwell test) in SRA01/04 cells [36].

lncRNAs affect the basic cellular processes of growth and division and are immediately associated with the survival and proliferation of lens epithelial cells in a diabetic state [32, 36]. NEAT1 silencing in HG-treated cells reduces cell viability and increases apoptosis in lens epithelial cells [32]. XIST silencing inhibits proliferation in HG-treated cells, as analyzed by an MTT assay [36]. PVT1 silencing relieves HG-induced inhibition of proliferation, as assessed by CCK-8 and EdU assays [37]. In addition, MALAT1 silencing in HG-treated cells alters the cell cycle profile, increasing the percentage of cells in the S phase [38].

Moreover, outside of the immediate ceRNA network, lncRNAs have been shown to participate in vast regulatory networks, such as those involving epitranscriptomics, to promote the stability of pathogenic mRNA transcripts (Yang [44]). METTL3 is increased in DC tissues and in HG-induced HLECs, associated with increased total m^6^A modification level. In functional studies, METTL3 knockdown increased proliferation and inhibited apoptosis in HG-induced HLECs. Furthermore, METTL3 bound explicitly to the 3'UTR of ICAM-1 mRNA to introduce m^6^A modifications, thereby promoting its stability and expression and contributing to the pathogenic state (Yang [44]). METTL3-mediated m⁶A modification of SIRT1 mRNA also contributes to diabetic cataract progression through altered autophagy and cellular senescence [49].

### Genome-wide transcriptomic dysregulation in DC

There was extensive reprogramming of the lens epithelial transcriptome in DC, with 8,326 lncRNAs dysregulated compared to non-diabetic controls [31]. Additionally, high glucose concentrations regulate disease-related lncRNAs such as MALAT1 and GAS5 [30, 33]. MALAT1 increases oxidative stress and apoptosis of lens epithelial cells cultured under high-glucose conditions, partly through the regulation of reactive oxygen species [38]. Moreover, lncRNA FOXD3–AS1 knockdown reduces oxidative damage by increasing the activity of antioxidant enzymes [43]. In addition, downregulation of lncRNA NEAT1 increases cell death and decreases cell growth [32].

LINC01508, MAFA-AS1, and MIAT functioned as central regulators associated with SIRT2. Additionally, there was an enhanced expression of lncRNAs, including MIAT [31]. In addition, elevated METTL3 levels stabilize disease-causing mRNAs, including ICAM1, thereby introducing post-transcriptional gene control to genomic alterations in DC (Yang [44]). Moreover, XIST, PVT1, and MALAT1 regulate proliferation, migration, apoptosis, and EMT [36–38].

## Meta-analysis results

### Expression

**Fig. 3:**
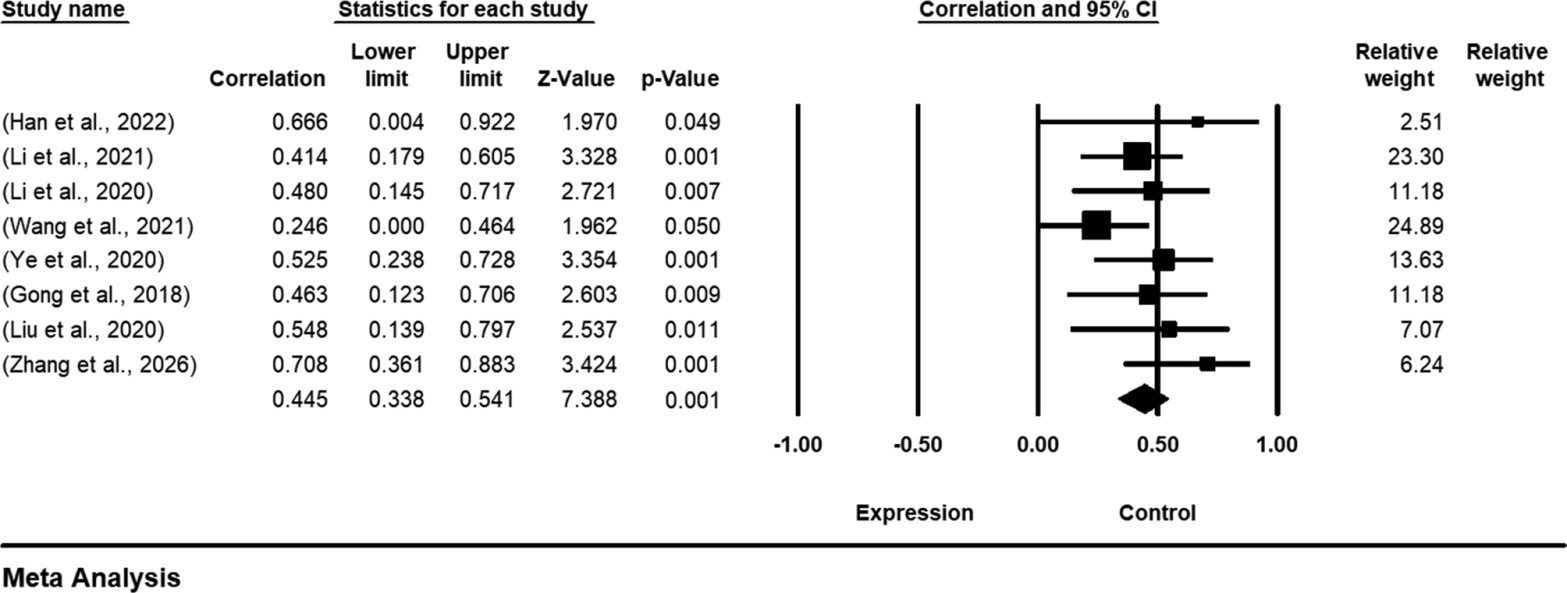
Forest plot of expression in DC-related lncRNA and lncRNA-linked epitranscriptomic datasets [30–34, 36]; [38, 46]

Data from eight datasets were included in the meta-analysis of expression in DC patients. LINC01508, MAFA-AS1, MIAT [31], GAS5 [33], XIST [36], KCNQ1OT1 [34], METTL16 [46], and MALAT1 [30, 38] were upregulated in DC-related samples, while NEAT1 was downregulated in the YY1/NEAT1/miR-205-3p/MMP16 pathway [32]. The random-effects model showed a pooled correlation r of 0.445 (95% CI: 0.338 to 0.541), *p* = 0.001 (Fig. 3). Statistical heterogeneity was low (I^2^ = 1.2%, Q = 7.088, df = 7, Q-test *p* = 0.42, τ^2^ = 0.00037, τ = 0.019), with a 95% prediction interval from 0.308 to 0.563. Biological heterogeneity remained clinically relevant as the analysis combined different lncRNAs, tissue sources, cell lines, comparator groups, assays, and glucose concentrations. Leave-one-out sensitivity analysis generated pooled correlations ranging from 0.422 to 0.502, supporting the stability of the pooled expression result. Exploratory year-based meta-regression did not materially change the expression association (slope = 0.035 on Fisher-z scale, *p* = 0.407).

**Fig. 4:**
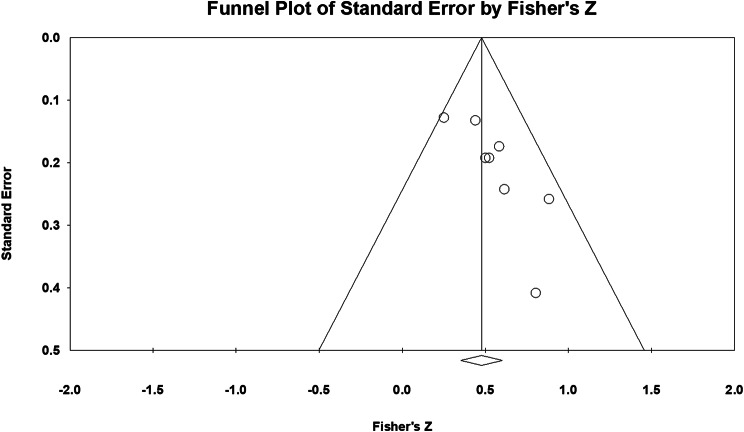
Funnel plot used to assess reporting bias in expression datasets in DC [30–34, 36]; [38, 46]

The corresponding funnel plot was used to assess reporting bias in expression datasets. Egger’s test was performed as an exploratory small-study-effect analysis and produced an intercept of 2.49 (*p* = 0.014). Funnel-plot and Egger-test findings were interpreted descriptively as fewer than 10 studies were included in this synthesis (Fig. 4).

## Discussion

The study examined the role of long non-coding RNAs in the pathogenesis of DC. It showed that there is a complex regulatory network in which certain lncRNAs coordinate dysfunction in lens epithelial cells through pathways related to apoptosis, oxidative stress, EMT, mitochondrial dysfunction, epitranscriptomic regulation, and impaired proliferation [9, 35]; Yang [37, 44]. Several lncRNAs are significantly dysregulated in DC tissues as compared to controls [31–33, 36]; [38].

SP1 expression was increased under high-glucose conditions, which promotes the expression of MALAT1 and PVT1 via direct promoter binding [30, 37]. Decreased NEAT1 expression is caused by YY1 downregulation in diabetic states [32]. SP1 is similarly implicated as a glucose-responsive transcription factor in diabetic nephropathy [50]. This indicates that glucose-sensitive transcription factors are important intermediates linking metabolic disturbance to epigenetic reorganization in lens epithelial cells. lncRNAs also induce pathogenic activity in DC through competing endogenous RNA (ceRNA) networks. Various lncRNAs function as molecular sponges that trap microRNAs, thereby alleviating post-transcriptional repression of target mRNAs and triggering downstream pathological pathways [51]. For instance, GAS5 silences miR-204-3p, resulting in TGFBR1 upregulation and activation of TGF-β/Smad signaling, which induces EMT (Li et al., 2021). Similarly, MALAT1 interacts with miR-144-3p, which suppresses NRF2 and activates the Notch1/Snail pathway, stimulating EMT and cellular migration [38]. XIST functions by absorbing miR-34a to increase SMAD2 expression, increase proliferation, and decrease apoptosis [52].

The overlapping lncRNA-miRNA-mRNA triad, exerting shared downstream effectors, such as the TGF-β/Smad pathway, indicates functional redundancy that ensures robust stimulation of pathological processes despite variability in molecular perturbation [53]. Such redundancy can also influence therapeutic targeting, as silencing a single lncRNA does not consistently avert disease progression when compensatory pathways remain.

In addition, lncRNAs influence oxidative stress by regulating redox homeostasis and cellular apoptotic vulnerability [54]. High-glucose-induced MALAT1 overexpression enhanced oxidative damage, as measured by malondialdehyde levels, and inhibited antioxidant enzymes such as superoxide dismutase and glutathione peroxidase [30]. This was a pro-oxidant effect mediated by activation of the p38MAPK signaling pathway, which induced apoptotic cascades, with increased Bax and caspase-3 expression and reduced Bcl-2 levels. In contrast, knockdown of FOXD3–AS1 relieved oxidative stress caused by high glucose by increasing miR-338-3p, thereby promoting antioxidative enzyme activity and decreasing apoptotic levels [43]. The protective role of FOXD3–AS1 underscores the potential of targeting specific lncRNAs to restore cellular homeostasis [55].

EMT involves reprogramming of the pathological process in which lens epithelial cells de-differentiate into mesenchymal cells, thereby increasing their migratory and invasive abilities [56]. Various lncRNAs, such as GAS5 and MALAT1, accumulate in the direction of EMT-stimulating mechanisms [33, 38]. GAS5 knockdown restored the epithelial marker E-cadherin, suppression of mesenchymal markers such as N-cadherin, vimentin, and α-smooth muscle actin, and suppression of cellular migration [33]. The mechanistic association of these phenotypic changes with inhibition of Smad2 phosphorylation suggested that GAS5 enhances EMT by stimulating canonical TGF-β signaling [33]. MALAT1 also induced EMT through the NRF2/Notch1/Snail axis and showed that a variety of lncRNAs can independently induce EMT via non-overlapping pathways [38]. Comparable MALAT1-driven EMT is reported in other high-glucose-exposed ocular epithelia, including retinal pigment epithelial cells [57, 58], and MALAT1 is similarly recognized as a key EMT regulator in oncological contexts [59].

The NEAT1/miR-205-3p/MMP16 axis and the PVT1/miR-214-3p/MMP2 axis were both involved in extracellular matrix remodeling and cellular invasion [32, 37]. This relates dysregulation of lncRNAs to cell phenotypic alterations, as well as to changes in the lens’s microenvironment, and how these can propagate the disease through two-way epithelial-stromal interactions.

In addition, lncRNAs are involved in higher-order regulation, such as epitranscriptomic changes [60]. METTL3 played a role in regulating pathogenic mRNA transcripts in lens epithelial cells (Yang [44]). Furthermore, METTL3 added m^6^A mRNA modifications to the 3'UTR of ICAM-1, which augmented its stability and expression, thereby facilitating cellular damage in high-glucose settings (Yang [44]). Moreover, the level of m^6^A modification was substantially higher in DC lens epithelial cells, and early fiber cells exhibited particularly close relationships between cataract severity and m^6^A dysregulation [45]. METTL16 regulated DKK1-mediated Wnt/β-catenin signaling in high-glucose-induced HLECs, while FTO-mediated m^6^A demethylation of lncRNA RMRP was linked to mitochondrial dysfunction in DC [35, 46]. METTL3 similarly regulates lens epithelial cell differentiation during normal lens development [61].

Epitranscriptomic dysregulation contributes to diabetic cataract pathogenesis. Increased expression of m^6^A regulators, particularly METTL3, together with elevated global m^6^A modification levels in diabetic lens epithelial cells. METTL3-mediated m^6^ A modification enhanced ICAM-1 expression and lens epithelial cell injury, while dysregulation of other m^6^A regulators, including METTL14, WTAP, FTO, ALKBH5, and YTHDF family proteins, further supports a role for epitranscriptomic remodeling in diabetic cataract.

### Study strengths and interpretive context

A combination of expression profiling and mechanistic functional data provides a multidimensional view of the role of lncRNAs in diabetic cataract, beyond expression-to-causation relationships in experimental manipulation studies. The mechanistic insights into particular lncRNA axes, such as MALAT1/miR-144-3p/NRF2, GAS5/miR-204-3p/TGFBR1, XIST/miR-34a/SMAD2, PVT1/miR-214-3p/MMP2, and NEAT1/miR-205-3p/MMP16, provide insights into the dysregulation and various pathological events. Additionally, the use of a wide range of methodological approaches, such as the quantitative RT-PCR, dual-luciferase reporter assays, RNA immunoprecipitation, chromatin immunoprecipitation, and functional cellular assays (apoptosis, proliferation, migration, EMT markers), enhances the reliability of the reported mechanisms.

The current evidence base was centered in China and was derived from experimental human tissue and high-glucose cell models, allowing coherent mechanistic mapping across genetic background, sampling procedures, and laboratory platforms. Individual studies used focused mechanistic designs that prioritized pathway interrogation. Different lncRNAs, experimental models, cell lines, glucose concentrations, comparator groups, and analytical platforms provide broad mechanistic coverage across DC-relevant biological processes while requiring outcome-specific interpretation of the pooled estimate despite the apparent statistical consistency.

### Study implications

The findings indicate that dysregulated lncRNAs are involved in multiple molecular pathways associated with diabetic cataract pathogenesis, including apoptosis, oxidative stress, EMT, mitochondrial dysfunction, epitranscriptomic regulation, and altered cellular proliferation. These results provide mechanistic insights into disease development and identify potential molecular targets for future investigation. The current evidence is derived predominantly from ex vivo tissue analyses and in vitro cell models, with clinical validation representing the next translational step. Therefore, the potential diagnostic, prognostic, or therapeutic relevance of these lncRNAs should be evaluated in well-designed clinical studies before clinical application.

METTL3-mediated m^6^A modification contributes to diabetic cataract pathogenesis and represents a potential epitranscriptomic therapeutic target. Prospective validation for cataract grading, disease progression, surgical outcomes, and non-invasive biomarker applications would advance clinical translation of the identified lncRNA and m^6^A-regulatory axes. Current evidence from lens tissues, cell models, and transcriptomic studies provides a structured foundation for prospective validation.

### Future research and knowledge gaps

There is a need to carry out clinical validation testing to determine the relationship between molecular dysregulation and clinical disease progression by longitudinally following cohorts of patients and assessing lncRNA expression. To map the evolutionary trend in lncRNA, such studies need to use standardized cataract grading systems and sample at different time points. Functional analyses using more advanced in vivo models, such as diabetic animal models and ex vivo human lens organ cultures, would provide useful information on the role of lncRNA in physiologically relevant settings more closely related to human disease.

Mechanistic investigations to elucidate the upstream cues that mediate changes in lncRNA expression in response to hyperglycemia would identify additional therapeutic intervention points and clarify whether lncRNA dysregulation is a primary response to metabolic stress or an aftereffect of other pathological events.

Studies that integrate lncRNA expression data with proteomic, metabolomic, and clinical phenotypic data would provide a more comprehensive view of DC pathophysiology and can identify new regulatory interactions not evident in RNA-based studies. Interactions across multi-omics would help reveal master regulators that organize multiple pathological pathways and are the best targets for therapeutic intervention. This broad-based molecular profiling would also enable stratification of patients for precision medicine and prediction of individual treatment response based on their specific molecular signature.

## Conclusions

This study describes lncRNAs as essential regulatory molecules in DC pathogenesis. Several lncRNAs were upregulated in DC compared to controls, while NEAT1 was downregulated in the YY1/NEAT1/miR-205-3p/MMP16 pathway and lncRNA-related m^6^A regulators showed coordinated epitranscriptomic dysregulation. Certain lncRNAs, such as MALAT1, NEAT1, PVT1, XIST, GAS5, KCNQ1OT1, FOXD3–AS1, and RMRP, orchestrate lens epithelial cell dysfunction via competing endogenous RNA networks and m^6^A-linked regulatory pathways, thereby triggering pathological signaling cascades of apoptosis, oxidative stress, EMT, mitochondrial dysfunction, and altered proliferation. The dysregulated lncRNAs suggest widespread transcriptomic reprogramming beyond the proven targets alone. These findings harmonize mechanisms previously studied separately into a coordinated molecular platform spanning transcriptional, post-transcriptional, and epitranscriptomic scales. The dependable patterns of dysregulation indicate their potential as candidate diagnostic biomarkers requiring validation and possible therapeutic targets for future preclinical testing. Future research should prioritize clinical validation studies, in vivo mechanistic investigations, multi-omics integration, and preclinical therapeutic development to translate these molecular insights into clinically effective strategies for preventing DC and reducing associated visual disability burden.

## Data Availability

All data generated or analyzed in this study are fully contained within the main manuscript and its tables and figures. No supplementary files were created or required for this systematic review and meta-analysis; therefore, no additional datasets are available.

## Abbreviations

α-SMA: Alpha-Smooth Muscle Actin
ALKBH5: AlkB Homolog 5
ARC: Age-Related Cataract
CAT: Catalase
CCK-8: Cell Counting Kit-8
ceRNA: Competing Endogenous RNA
ChIP: Chromatin Immunoprecipitation
CI: Confidence Interval
DC: Diabetic Cataract
DNA: Deoxyribonucleic Acid
EdU: 5-Ethynyl-2′-deoxyuridine
EMT: Epithelial-Mesenchymal Transition
FISH: Fluorescence In Situ Hybridization
FTO: Fat Mass and Obesity-Associated Protein
GAS5: Growth Arrest Specific 5
GO: Gene Ontology
GSH-Px: Glutathione Peroxidase
GRADE: Grading of Recommendations Assessment, Development and Evaluation
HG: High Glucose
HLE B-3: Human Lens Epithelial B-3 Cell Line
HLEC: Human Lens Epithelial Cell
ICAM-1: Intercellular Adhesion Molecule 1
JBI: Joanna Briggs Institute
KEGG: Kyoto Encyclopedia of Genes and Genomes
LEC: Lens Epithelial Cell
lncRNA: Long Non-Coding RNA
m^6^A: N6-Methyladenosine
MAPK: – Mitogen-Activated Protein Kinase
MDA: Malondialdehyde
METTL3: Methyltransferase-Like 3
METTL14: Methyltransferase-Like 14
METTL16: Methyltransferase-Like 16
MMP: Matrix Metalloproteinase
MTT: Methylthiazolyldiphenyl-Tetrazolium Bromide
NA: Not Applicable
NG: Normal Glucose
NRF2: Nuclear Factor Erythroid 2-Related Factor 2
NR: Not Reported
PICOS: Population, Intervention, Comparison, Outcome, Study Design
PRISMA: Preferred Reporting Items for Systematic Reviews and Meta-Analyses
PROSPERO: International Prospective Register of Systematic Reviews
qRT-PCR: Quantitative Reverse Transcription Polymerase Chain Reaction
RIP: RNA Immunoprecipitation
RNA: Ribonucleic Acid
ROS: Reactive Oxygen Species
RT-qPCR: Reverse Transcription Quantitative Polymerase Chain Reaction
SMAD: Small Mothers Against Decapentaplegic
SOD: Superoxide Dismutase
SMD: Standardized Mean Difference
SP1: Specificity Protein 1
SRA01/04: Human Lens Epithelial Cell Line SRA01/04
T2DM: Type 2 Diabetes Mellitus
TGF-β: Transforming Growth Factor-Beta
TGFBR1: Transforming Growth Factor-Beta Receptor 1
TUNEL: Terminal Deoxynucleotidyl Transferase dUTP Nick-End Labeling
WTAP: Wilms Tumor 1-Associated Protein
XIST: X-Inactive Specific Transcript
YY1: Yin Yang 1
YTHDF: YTH N6-Methyladenosine RNA Binding Protein Family

## Declarations

### Integrity and Transparency

The authors confirm that no artificial intelligence was used in the generation or submission of this manuscript.

### Guarantor

C.-M. C. (Chi-Ming Chan) is the guarantor of this work and accepts full responsibility for the integrity of the study, the accuracy of the data analysis, and the decision to submit the manuscript for publication. The guarantor had full access to all extracted data and takes responsibility for the overall content of the manuscript.
